# Translation, adaptation, and validation of the body image scale among cancer patients in Malaysia

**DOI:** 10.3389/fpubh.2026.1797807

**Published:** 2026-03-24

**Authors:** Wenjun Song, Nor Shuhada Mansor, Ruiling Zhang, Mohammad Farris Iman Leong Bin Abdullah, Jian Zhan

**Affiliations:** 1 The Second Affiliated Hospital of Zunyi Medical University, Zunyi, Guizhou, China; 2 Department of Community Health, Advanced Medical and Dental Institute, Universiti Sains Malaysia, Kepala Batas, Pulau Pinang, Malaysia; 3 Second Affiliated Hospital of Xinxiang Medical University, Xinxiang, Henan, China; 4 Department of Psychiatry and Mental Health, Faculty of Medicine, Universiti Sultan Zainal Abidin, Kuala Terengganu, Malaysia

**Keywords:** body image scale, cancer patients, Malay, reliability, validity

## Abstract

**Background:**

Changes in body image resulting from cancer and its treatment’s adverse effects negatively impact mental health and quality of life. However, no instrument to measure body image concerns has been translated, adapted, and validated for cancer patients in Malaysia. This study translated the Body Image Scale (BIS) into Malay and assessed its psychometric properties among patients with various types of cancer in Malaysia.

**Methods:**

Initially, the translation of the original English version of the BIS was conducted by two native Malay-speaking translators and back translation by a native English-speaking translator. The Malay version of the Body Image Scale (BIS-M) was then evaluated by a panel of content experts to attain content validity and then tested in a pilot study with 30 native Malay-speaking cancer patients to attain face validity. Then, 200 cancer patients with various types of cancer were recruited, and they were administered the sociodemographic and clinical characteristics questionnaire, the BIS-M, and the Malay versions of the Body Self-Image Questionnaire-Short Form (BSIQ-SF) (as comparator to assess convergent validity) and Acceptance and Action Questionnaire version II (AAQ II-M) (as comparator to assess discriminant validity). The internal consistency, test–retest reliability, construct validity, and convergent and discriminant validity of the BIS-M were evaluated.

**Results:**

The BIS-M exhibited good internal consistency with Cronbach’s *α* of 0.895 and good test–retest reliability with the intraclass correlation coefficient of its items, and the total score ranged from 0.605 to 0.885 (*p* < 0.001). The BIS-M demonstrated good face, content, convergent, and discriminant validity. Construct validity assessment with confirmatory factor analysis (CFA) revealed that BIS-M consisted of a single factor similar to the original English version [chi square to degree of freedom ratio (*χ*^2^/df) = 1.862, *p =* 0.004; comparative fit index (CFI) = 0.974, goodness of fit index (GFI) = 0.935, Tucker Lewis index (TLI) = 0.959, root mean square of error of approximation (RMSEA) = 0.056].

**Conclusion:**

The BIS-M is ready for use to assess body image issues which affect cancer patients of both genders. This instrument is expected to evaluate the effectiveness of various psychosocial interventions on enhancing positively perceived body image among cancer patients in Malaysia.

## 1 Introduction

Cancer is a challenging disease that leads to negative psychological effects due to its symptoms and treatment side effects. It is perceived as an ordeal by patients and their families, significantly affecting both their physical and emotional wellbeing (1). Among cancer patients, the detrimental impact on body image is recognized as a significant psychosocial issue (2). Changes in body image are a major concern for many cancer survivors (3). Body image is a complex concept that extends beyond the mere perception of physical appearance (4). It is often described as a multifaceted construct encompassing perceptions, thoughts, emotions, and behaviors related to the entire body and its functioning (5).

Body image is associated with interoception, which refers to the perception of internal bodily sensations, such as feeling hunger due to an empty stomach, thirst due to a dry mouth, or a fast heartbeat associated with anxiety. Interoception forms the foundation of body image—when bodily sensations are perceived as safe and satisfactory, individuals tend to have a more positive body image. Conversely, low interoceptive awareness and satisfaction may lead to body image dissatisfaction and related concerns (6, 7). Functional interoception and a positive body image have been shown to enhance quality of life among cancer patients (8).

Breast cancer is one of the most common types of cancer worldwide. Psychological complications are highly prevalent among breast cancer survivors and can substantially affect their mental wellbeing, with body image dissatisfaction being one of the most frequent issues (9). Several psychological treatments have demonstrated efficacy in maintaining and restoring the psychological wellbeing of breast cancer patients (10). However, there is a lack of data on the effectiveness of these psychological interventions in improving positive body image and interoceptive awareness and satisfaction among cancer patients. Therefore, there is a need for an instrument that can measure the perception and acceptance of body image changes in cancer patients and evaluate how psychological treatments may enhance these aspects.

Several instruments assess the perception and acceptance of body image changes, including the Body Shape Questionnaire-34 (BSQ), the Body Image Questionnaire-20 (FKB-20), the Body Image-Acceptance and Action Questionnaire (BI-AAQ), and the Body Image Scale (BIS). The BSQ comprises 34 items (11), while the FKB-20 consists of 20 items (12), both of which require longer administration time and may affect concentration, particularly in cancer patients experiencing symptoms or treatment side effects. The BI-AAQ contains 12 items measuring body image flexibility and acceptance of bodily changes, validated among individuals with eating disorders (13). Conveniently, the 10-item BIS assesses emotional (e.g., feeling self-conscious), behavioral (e.g., difficulty looking at oneself when nude), and cognitive (e.g., satisfaction with appearance) aspects of body image in cancer patients (14). Although it has the fewest items among these instruments, the BIS remains comprehensive, capturing affective, behavioral, and cognitive components of body image change (14). Hence, the BIS is suitable for assessing acceptance of body image change in cancer patients, as its shorter administration time may minimize response bias caused by physical discomfort. This confers a pivotal role to the BIS to assess the efficacy of psychological intervention that may enhance body image among cancer patients. For example, **acceptance and commitment therapy** (ACT) may be effective to facilitates the acceptance of the negative emotions, behaviors and cognitive distortion associated with body image of cancer patients and enhance self compassion in relation to body image (15) which would be sufficiently measure with the BIS in intervention studies. Similarly, **mindfulness-based stress reduction** (MBSR) may also induce similar effects by fostering the non-judgmental experience of these negative impacts of cancer on body image (16) which could be easily assess with the BIS to investigate the efficacy of MBSR to ameliorate negative body image perception.

The items in the original English version of the BIS were developed through literature review, discussions with healthcare professionals, and extensive interviews with breast cancer patients. Initially, 10 items were created, covering affective (e.g., feeling feminine, feeling attractive), behavioral (e.g., difficulty looking at oneself when naked, avoiding people due to appearance), and cognitive (e.g., satisfaction with appearance or scar) domains. The initial test on 276 cancer patients (14) revealed discomfort with positively phrased items, leading to a revision in which all items were rephrased negatively. The revised BIS underwent psychometric testing in 682 breast cancer patients, demonstrating excellent internal consistency (Cronbach’s *α* = 0.93). Its clinical validity was supported by good discriminant validity, high sensitivity, and consistent scores across multicenter settings. Factor analysis using the General Least Squares method indicated that the BIS consists of a single factor explaining 57.55, 50.18, and 53.05% of the variance in three separate analyses (14).

The BIS has shown good psychometric properties across various cancer types, including head and neck, colorectal, and breast cancers (14, 16–18). It has been translated into multiple languages—Greek, Spanish, Turkish, Korean, Portuguese, Dutch, Thai, and Chinese—and has consistently demonstrated reliability and validity in diverse cultural contexts (19–26). These findings confirm that the BIS is an effective tool for assessing body image disturbances in cancer patients, making it suitable for both clinical trials and patient care.

In Malaysia, several body image scales, such as the Multidimensional Body Image Scale and the Body Appreciation Scale-2, have been validated; however, they do not fully capture the specific body image concerns of adult cancer patients (27–29). The Body Self-Image Questionnaire-Short Form (BSIQ-SF), validated among young adults, focuses on physical functionality and attractiveness but is less relevant to illness-related body image changes (30). The BIS, however, is more suitable for assessing body image disturbances in cancer patients, although it has not yet been validated in Malaysia’s cancer population or translated into Malay.

It is crucial for researchers and clinicians to appropriately assess body image issues among Malay-speaking cancer patients, given the detrimental effects that body image changes can have on mental health outcomes. Therefore, this study sought to answer the research question: “Can the BIS be translated into Malay and adapted to assess acceptance of body image change among cancer patients in Malaysia?” We hypothesized that the Malay version of the BIS (BIS-M): (1) would demonstrate good face and content validity, (2) would show good reliability through high internal consistency and test–retest reliability, and (3) would exhibit good construct validity, evidenced by strong convergent and discriminant validity, with CFA confirming a unidimensional structure similar to the original English version. To address this research gap, the present study aimed to: (1) translate the original English version of the BIS into Malay, (2) determine the reliability of the BIS-M through internal consistency and test–retest reliability, and (3) evaluate the validity of the BIS-M by assessing its face, content, convergent, and discriminant validity, as well as its CFA results among cancer patients in Malaysia.

## 2 Materials and methods

### 2.1 Translation process, face and content validity

#### 2.1.1 Translation and back translation

Data were collected as previously described in Song et al. (54, Jan 13) in their validation study of the Malay version of the Illness Cognition Questionnaire among cancer patients in Malaysia. Because there was no existing BIS-M, the BIS first needed to be translated into Malay. The translation and adaptation process followed the International Test Commission Guidelines for Translating and Adapting Tests (2nd Edition) (International Test Commission).

The process began with preparation and planning, forming a team of experts, and establishing translation guidelines. Two independent native Malay-speaking bilingual translators translated the BIS into Malay, carefully considering cultural nuances. Both translators were clinical psychologists (holding doctoral degrees in clinical psychology), and one of their research areas was psycho-oncology.

Next, a native English-speaking bilingual translator who had not seen the original English version of the BIS performed the back translation of the Malay version. This translator was a psychiatrist (holding a postgraduate clinical qualification in psychiatry) with research experience in psycho-oncology.

#### 2.1.2 Assessing content validity

Data were collected as previously described in Song et al. (54, Jan 13) in their validation study of the Malay version of the Illness Cognition Questionnaire among cancer patients in Malaysia. A panel of experts from **Universiti Sains Malaysia (USM)** and Universiti Sultan Zainal Abidin was invited via email to participate in a face-to-face review of the forward and backward translations of the BIS-M.

The eligibility criteria for the experts included:

1) An academic qualification of at least a Master of Medicine or a doctoral degree in a related field such as mental health, community health, or public health; and.
2) At least 5 years of research experience after completing their highest postgraduate qualification in an area related to mental health, body image research, or cross-cultural translation and validation of questionnaires.

The expert panel comprised a psychiatrist, two clinical psychologists, and three public health/community health specialists who reviewed the back-translated version to ensure accuracy and cultural relevance. This expert panel produced a draft of the BIS-M. The psychiatrist and two psychologists were not the same individuals who served as translators of the BIS-M. The characteristics of the expert panel are presented in Table 1.

**Table 1:** The characteristics of the expert panel in this study.

Expert panel	Post	Qualification	Occupation	Experience	Role in study
Expert panel 1	Psychiatrist	Doctor of Psychiatry	Senior medical lecturer at a public university in Malaysia	10 years of research work in psycho-oncology and cross-cultural validation of questionnaires	Forward translation, Content validity
Expert panel 2	Clinical psychologist	PhD in psychology	Lecturer at a public university in Malaysia	5 years of research work in psycho-oncology and cross-cultural validation of questionnaires	Forward translation, Content validity
Expert panel 3	Clinical psychologist	PhD in psychology	Lecturer at a public university in Malaysia	5 years of research work in psycho-oncology and cross-cultural validation of questionnaires	Back translation, Content validity
Expert panel 4	Community health specialist	PhD in community health	Senior lecturer at a public university in Malaysia	More than 5 years of research work in cancer and cross-cultural validation of questionnaires	Content validity
Expert panel 5	Community health specialist	PhD in community health	Senior lecturer at a public university in Malaysia	More than 5 years of research work in cancer and cross-cultural validation of questionnaires	Content validity
Expert panel 6	Public health specialist	PhD in public health	Senior medical lecturer at a public university in Malaysia	10 years of research work in cancer epidemiology and cross-cultural validation of questionnaires	Content validity

The indices used to assess content validity included the item-level content validity index (I-CVI), the scale-level content validity index based on universal agreement (S-CVI/UA), and the average scale-level content validity index (S-CVI/Ave). Each expert rated the relevance of each BIS-M item in assessing perceived body image among cancer patients using the following scale:

(1) not relevant, (2) partially relevant, (3) relevant, or (4) very relevant.

Each rating of 3 or 4 was assigned a score of 1, while ratings of 1 or 2 were assigned a score of 0 (31). The I-CVI was calculated by dividing the number of experts who assigned a score of 1 to an item by the total number of experts. An item’s universal agreement (UA) was scored as 1 if all experts rated it 3 or 4; otherwise, it was scored 0. The S-CVI/UA was computed by dividing the total number of items with universal agreement by the total number of items. The S-CVI/Ave was calculated as the sum of all I-CVI values divided by the total number of items.

To establish that the BIS-M achieved satisfactory content validity, the I-CVI for each item had to exceed 0.83, the S-CVI/UA had to be greater than 0.8, and the S-CVI/Ave had to be greater than 0.9 (31).

#### 2.1.3 Assessing face validity

A pilot study was conducted to assess face validity. Participants were recruited from the source population—cancer patients receiving treatment at the Oncology Clinic, Radiotherapy Unit, and Daycare Department of the **Advanced Medical and Dental Institute (AMDI)**, USM. All participants met the study’s eligibility criteria. Recruitment was conducted by a research assistant who was not part of the research team and was not aware of the objectives of the study. The research assistant held a bachelor degree in psychology and received training in subject recruitment and data collection for 1 week prior to subject recruitment. A total of 30 native Malay-speaking cancer survivors were recruited for the pilot study (32).

Each participant completed the BIS-M and was subsequently interviewed to provide feedback on clarity, comprehensibility, administration time, cultural appropriateness, and the semantic quality of the instructions, items, and response format. Participants rated each item’s clarity and comprehensibility on a 4-point scale:

1 = “not clear or understandable,”

2 = “somewhat clear or understandable,”

3 = “clear and understandable,” and.

4 = “very clear and understandable.”

Responses of 3 or 4 were considered as agreement with the clarity and comprehensibility of the item. The item-level face validity index (I-FVI) was calculated as the number of agreed responses divided by the total number of raters. The average scale-level face validity index (S-FVI/Ave) was calculated as the sum of I-FVI values across all items divided by the total number of items. The S-FVI/UA was calculated as the number of items with universal agreement (all raters rated 3 or 4) divided by the total number of items. FVI values were considered acceptable if ≥ 0.83 (32).

### 2.2 Study design and participants

This validation study was conducted from April to June 2023. Ethical approval was granted by the Human Research Ethics Committee of USM (code: USM/JEPeM/22080569). Data were collected as partially described in Song et al. (54, Jan 13) in their validation study of the Malay version of the Illness Cognition Questionnaire among cancer patients in Malaysia.

A total of 200 participants with different types of cancer were recruited from the Oncology Clinic, Radiotherapy Unit, and Daycare Department of AMDI, USM by the same research assistant who conducted the subject recruitment in the pilot study. The source population was all cancer patients who registered for treatment in the Oncology unit of AMDI, USM. The research assistant approached the cancer patients during their clinic appointment in the oncology outpatient clinic and while they were admitted to daycare for treatment. As AMDI, USM is a referral center for oncology treatment in the northern region of Peninsular Malaysia, the patients came from a few states in the vicinity of AMDI. USM, such as Perlis, Kedah, Pulau Pinang and Perak.

Sample size calculations were based on different validation objectives:

For internal consistency, 137 subjects (including a 20% dropout rate) were required (33).

For convergent validity, 24 subjects were needed (including a 20% dropout rate) (34).

For discriminant validity, 154 subjects were required (including a 20% dropout rate) (35).

For CFA, 186 subjects (including a 20% dropout rate) were required.

A 20% dropout rate was added based on previous Malaysian studies involving cancer patients (35). Since the largest required sample size was for CFA, the final target sample size for this study was 186 participants.

Consecutive sampling was used, whereby participants meeting the eligibility criteria were recruited during the study period (36). Potential participants were screened based on the inclusion criteria:

1) A confirmed cancer diagnosis (any type, stage, or duration),
2) Aged 18 years or older, and.
3) Able to read and write in Malay.

Exclusion criteria included:

1) Being physically unfit to complete questionnaires (e.g., bedridden, comatose, confused, unable to speak, or too weak), and.
2) Being cognitively impaired.

All participants received a full explanation of the study objectives and confidentiality assurances before participation. Written informed consent was obtained from each participant.

### 2.3 Data collection

Participants completed the socio-demographic and clinical characteristics questionnaire, the BIS-M, and the AAQ-II-M.

#### 2.3.1 Socio-demographic and clinical characteristics questionnaire

Socio-demographic variables included age, gender, ethnicity, marital status, monthly household income, and education level. Clinical variables included cancer type, stage, and time since diagnosis. The questionnaire and its response options are presented in Supplementary Appendix 1. It was administered in Malay.

#### 2.3.2 Body image scale (BIS)

The BIS is a 10-item questionnaire designed to assess cancer patients’ perceptions of body image changes. Each item is rated on a 4-point Likert scale (0 = “not at all” to 3 = “very much”), with total scores ranging from 0 to 30. Higher scores indicate greater distress or body image concerns. The original version demonstrated a single-factor structure and good psychometric properties, including strong validity and reliability (Cronbach’s *α* = 0.93) (14).

#### 2.3.3 Body self-image questionnaire–short form (BSIQ-SF)

The BSIQ-SF is a self-administered tool assessing body image perception across nine domains, comprising 27 items. It offers advantages over the original version due to reduced response burden and improved response rate and data quality. Items are rated on a 5-point Likert scale ranging from “Not at all true of myself” to “Completely true of myself.” The BSIQ-SF has demonstrated good psychometric properties (37). The Malay version (BSIQ-SF-M) was validated among young adults in Malaysia, with exploratory and confirmatory factor analyses revealing 21 items across four domains (30). Permission to use the BSIQ-SF-M was obtained from the author (Supplementary Appendix 3).

#### 2.3.4 Acceptance and action questionnaire II (AAQ-II)

The AAQ-II is a self-reported measure of experiential avoidance—the tendency to avoid or suppress unpleasant internal experiences rather than accept them. It consists of seven items, and higher scores indicate greater psychological inflexibility (38). The Malay version (AAQ-II-M) was adapted and validated among cancer patients in Malaysia, showing a single-factor structure consistent with the original version and excellent internal consistency (Cronbach’s *α* = 0.91) (39). The AAQ-II-M was used to assess the discriminant validity of the BIS-M, as both instruments measure distinct constructs. Permission to use the AAQ-II-M is included in Supplementary Appendix 3.

### 2.4 Data analysis

Data were analyzed using the Statistical Package for the Social Sciences version 29 (SPSS v29; IBM Corp, Armonk, NY), and CFA was conducted using AMOS version 26 (IBM SPSS, Chicago). Descriptive statistics were reported for socio-demographic and clinical characteristics: categorical variables as frequencies and percentages, and continuous variables as means and standard deviations (SD).

Reliability was assessed through internal consistency using McDonald’s Omega (*ω*), with values ≥ 0.7 considered acceptable. Test–retest reliability was assessed by re-administering the BIS-M to 100 participants two weeks after the initial administration. As all participants had completed their cancer treatment, body image perceptions were not expected to change significantly over this interval. The intraclass correlation coefficient (ICC) between the two administrations was computed, with statistical significance set at *p* < 0.05.

Convergent validity was assessed using Pearson’s correlation between the BIS-M total score and the domain and total scores of the BSIQ-SF-M. Discriminant validity was examined using Pearson’s correlation between the BIS-M and AAQ-II-M total scores. Correlation coefficients were interpreted as weak (0–0.3), moderate (0.3–0.5), or strong (>0.5) (31).

CFA was conducted using the maximum likelihood estimator. In this study, we examined the 1-factor model suggested by the English version of the BIS and compared the model fitting of the 1-factor model with a 2-factor model of the BIS-M. If the best fitting model was the 1-factor model and not 2-factor model, this confirmed that the BIS-M factor structure was unidimensional and did not go beyond one factor. Model identification employed a marker indicator. The following fit indices were evaluated:

1) CFI,
2) TLI,
3) GFI,
4) RMSEA, and
5) *χ*^2^/df.

According to Hu and Bentler (55), acceptable cutoff values are: CFI and TLI ≈ 0.95, GFI ≈ 0.90, RMSEA ≈ 0.06, and *χ*^2^/df < 2.0 (40). Inter-item correlations were also examined, with values > 0.3 indicating acceptable internal reliability.

Then, a subgroup analysis was performed with independent *t*-test or one-way ANOVA to assess how well the BIS-M differentiate between the degree of body image between different subgroups of the demographic and clinical characteristics, such as gender, age, education status, religion, time since diagnosis, cancer types and cancer stage (41–43). Statistical significance was set at *p* < 0.05 and two-tailed.

## 3 Results

### 3.1 Translation and adaptation of the BIS-M

#### 3.1.1 Content validity index (CVI) of BIS-M

Table 2 provides a summary of the BIS-M’s content validity evaluation. The I-CVI of all the items of the BIS-M was one except for item 6, which was 0.83. Similarly, the UA of all the items was one except for item 6. The S-CVI/Ave was 0.98, while the S-CVI/UA was 0.90.

**Table 2:** Content validity index of the BIS-M.

Items	Expert 1	Expert 2	Expert 3	Expert 4	Expert 5	Expert 6	Expert in agreement	I-CVI	UA
Item 1	1	1	1	1	1	1	6	1	1
Item 2	1	1	1	1	1	1	6	1	1
Item 3	1	1	1	1	1	1	6	1	1
Item 4	1	1	1	1	1	1	6	1	1
Item 5	1	1	1	1	1	1	6	1	1
Item 6	1	1	1	0	1	1	5	0.83	0
Item 7	1	1	1	1	1	1	6	1	1
Item 8	1	1	1	1	1	1	6	1	1
Item 9	1	1	1	1	1	1	6	1	1
Item 10	1	1	1	1	1	1	6	1	1
Proportion relevance									
							S-CVI/Ave:	0.98	
							S-CVI/UA:		0.90

#### 3.1.2 The face validity of the BIS-M

The pilot research, which included 30 native Malay-speaking cancer patients, assessed the BIS-M’s face validity. The I-FVI for all the items ranged from 0.9 to 1.0. The S-FVI/Ave based on the I-FVI was 0.983, while the S-FVI/Ave based on proportion of clarity and comprehension was 0.983. Finally, the S-FVI/UA was at 0.9.

### 3.2 Participant characteristics

The socio-demographic and clinical characteristics, and the mean BIS-M score of the participants in this study are summarized in Table 3. The majority of participants were aged between 45–65 years (56.0%), and the sample predominantly comprised females (82.5%) and Malays (81.0%). Most participants reported a monthly household income categorized as the low-income group (B40) with a monthly income of less than RM6,400 or USD 1,450 (77.5%) and had attained secondary education (57.5%). A significant proportion of participants were married (84%) and primarily affected by breast cancer (51.5%). The distribution across stages of cancer varied, with the majority diagnosed at Stage 2 (41%) and the smallest proportion of patients in Stage 4 (8%) (questionnaires for use in cancer patients should also include patients in stage 4 in order to ensure that the validated questionnaire is appropriate for a wide range of cancer patients, including those with palliative care). More than half of the study participants were treated with surgery plus chemotherapy and chemotherapy plus radiotherapy (66%). The mean total score on the BIS-M was 20.50 (SD = 6.42).

**Table 3:** Socio-demographic and clinical characteristics, and the mean BIS-M score of the participants.

Variables	Number of participants (*n*)	Percentage (%)
Age		
18–25 years old	5	2.5
26–45 years old	55	27.5
45–65 years	112	56.0
> 65 years	28	14.0
Gender		
Male	35	17.5
Female	165	82.5
Ethnicity		
Malays	162	81.0
Chinese	26	13.0
Indian	10	5.0
Others	2	1.0
Monthly household income		
B40 (< RM 6,400/< USD 1,450)	155	77.5
M40 (RM 6,400-RM 11,000/USD 1,450 to USD 2,492)	42	21.0
T20 (> RM 11,000/> USD 2,492)	3	1.5
Education		
Primary or below	17	8.5
Secondary	115	57.5
tertiary	68	34.0
Marital status		
Married	168	84
Single/divorcee/widow/widower	32	16
Time since diagnosis		
< 3 months	39	19.5
3–6 months	42	21
6 month- 1 year	36	18
1–2 year	27	13.5
>2 years	56	28.0
Types of cancer		
Breast cancer	103	51.5
Lung cancer	4	2.0
Head and neck cancer	39	19.5
Colon cancer	17	8.5
Others	37	18.5
Stage of cancer		
Stage 1	26	13.0
Stage 2	82	41.0
Stage 3	76	38.0
Stage 4	16	8.0
Mode of treatment received		
Surgery + chemotherapy	76	38.0
Chemotherapy + radiotherapy	56	28.0
Surgery + chemotherapy + radiotherapy	45	22.5
Surgery + radiotherapy	13	6.5
Others	10	5.0
BIS-M score	20.50^a^	6.42^b^

B40 = the low-income group, M40 = the middle-income group, T20 = the high-income group. ^a^ = mean, ^b^ = standard deviation.

### 3.3 Confirmatory factor analyses (CFA)

**Figure 1:**
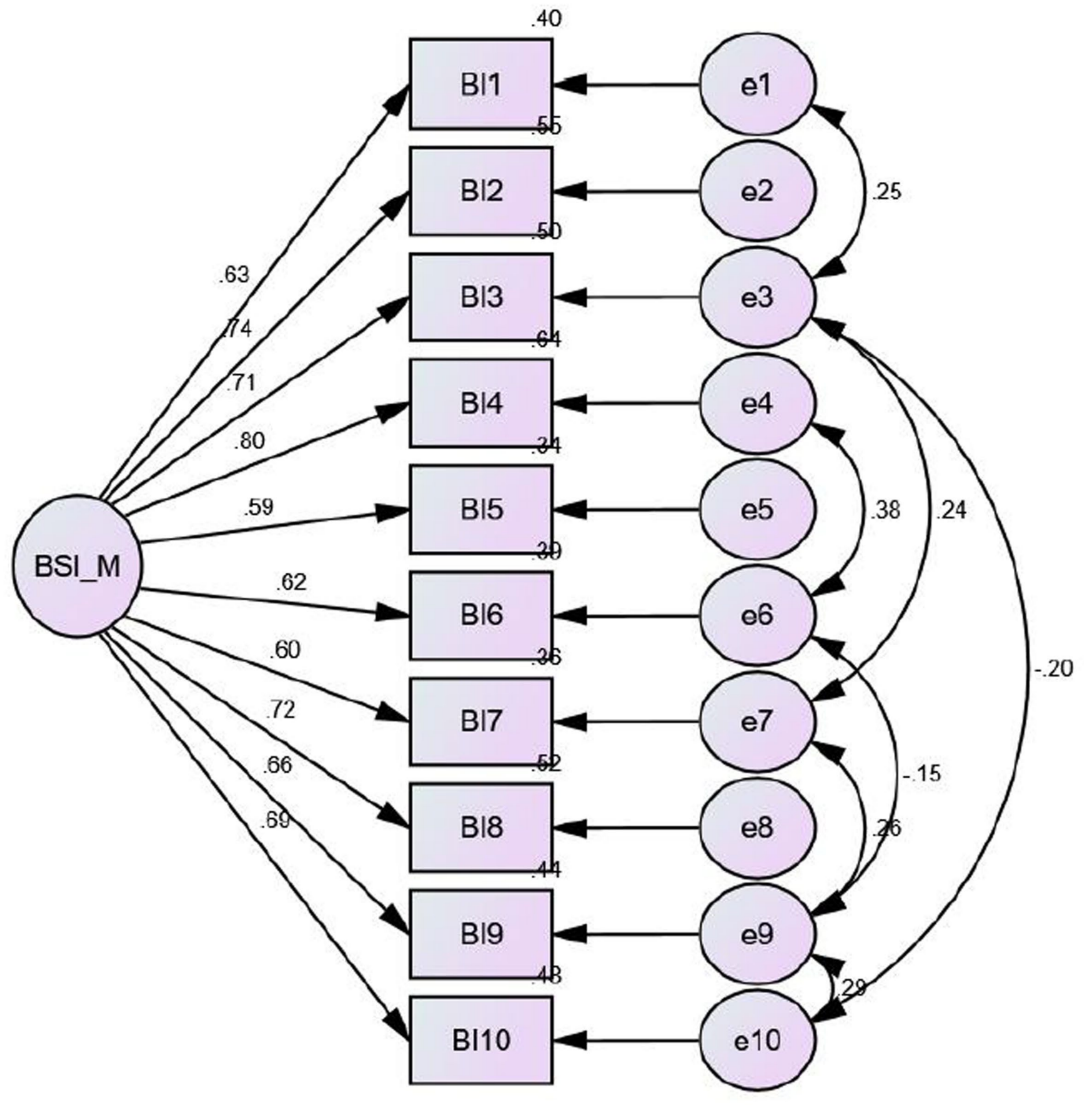
Structural equation model diagram showing a latent variable labeled BSI_M on the left, with arrows pointing to ten indicator variables Bl1 to Bl10, each with a factor loading from .59 to .80. Each indicator variable points to an error term to e10, with error variances from .30 to .55. Curved arrows show correlations among error terms, with coefficients ranging from −.20 to .38.

The CFA of the BIS-M is illustrated in Table 4 and Figure 1. Initially, the 2-factor model of BIS-M was not fitting (ჯ^2^/df = 2.925, *p* < 0.001, CFI = 0.933, GFI = 0.933, TLI = 0.908, RMSEA = 0.098). The 1-factor model of the BIS-M was the best-fitting model, which is similar to the finding in the original English version of the BIS-M (ჯ^2^/df = 1.862, *p =* 0.004, CFI = 0.974, GFI = 0.935, TLI = 0.959, RMSEA = 0.056). Since the chi-square/degree of freedom was < 2, CFI was > 0.95, TFI was > 9.5, GFI was > 0.90, and RMSEA was < 0.06, the 1-factor model of the BIS-M was fitting well. The average inter-item correlation was >0.3.

**Table 4:** Confirmatory factor analysis of the BIS-M.

Variables	1-factor model of the BIS-M	2-factor model of the BIS-M
Chi-square/degree of freedom (*ჯ*^2^/df)	1.862 (*p =* 0.004)	2.925 (*p* < 0.001)
Comparative fit index (CFI)	0.974	0.933
Goodness of fit index (GFI)	0.935	0.933
Tucker-Lewis index (TLI)	0.959	0.908
Root mean square error of approximation (RMSEA)	0.056	0.098

### 3.4 Convergent and discriminant validity of the BIS-M

The convergent validity of the BIS-M is summarized in Table 5. A higher correlation coefficient suggests that the items of the BIS-M was more closely related to the domains and total score of the BSIQ-SF-M, whereby the latter also measure the degree of body image. Our findings indicate that all the items of the BIS-M have significant and high correlation coefficient with the domains (correlation coefficient ranged from −0.746 to 0.779, *p* < 0.001) and total score of the BSIQ-SF-M (correlation coefficient = −0.821, p < 0.001; total score calculated from sum of the reverse scores of negative affect and height dissatisfaction, and direct scores of attractiveness evaluation and physical function awareness), demonstrating good convergent validity.

**Table 5:** Convergent validity of the Malay version of BIS.

BIS-M BSIQ-SF-M	Negative affect	Attractiveness evaluation	Physical functional awareness	Height dissatisfaction	Total score (after reversal of negative affect and height dissatisfaction scores)
Total score	0.779*	−0.746*	−0.692*	0.666*	−0.821*

*p < 0.001.

By looking at the correlations between the BIS items and another measure that evaluates a different construct, it is feasible to assess the BIS’s discriminant validity in the Malay language. The discriminant validity of the BIS-M is presented in Table 6. Our findings revealed that all the items of BIS-M and its total score had low or non-significant correlation coefficients with the total score of the AAQ-II-M (coefficient ranged from 0.001 to 0.151), indicating that the BIS-M had good discriminant validity. Lower correlations suggest that the BIS items measure a distinct construct compared to AAQ-II-M. The results show that the BIS is capturing a unique aspect of body image concerns separate from psychological flexibility as measured by the AAQ II.

**Table 6:** Discriminant validity of the Malay version of BIS.

BIS items	AAQ II total
Item 1	0.027
Item 2	0.116*
Item 3	0.041
Item 4	0.001
Item 5	0.015
Item 6	0.053
Item 7	0.136*
Item 8	0.151*
Item 9	0.050
Item 10	0.038
BIS total	0.075

*p < 0.05.

### 3.5 Reliability

#### 3.5.1 Internal consistency

The overall score’s Cronbach’s alpha value was 0.895, while the McDonald omega of the total BIS-M score was at 0.893. There was not much difference between the internal consistency of BIS-M assessed using Cronbach’s alpha and McDonald omega. Hence, we can conclude that the BIS-M had a good internal consistency.

#### 3.5.2 Test–retest reliability

Table 7 provides a summary of the BIS-M’s test–retest reliability. All the items of the BIS-M and its total score exhibited a high intraclass correlation coefficient ranging from 0.605 to 0.885, indicating excellent test–retest reliability for the BIS-M.

**Table 7:** Test–retest reliability of the BIS-M.

BIS items	Test–retest reliability (intraclass correlation coefficient)
Item 1	0.605*
Item 2	0.851*
Item 3	0.804*
Item 4	0.853*
Item 5	0.736*
Item 6	0.806*
Item 7	0.802*
Item 8	0.877*
Item 9	0.853*
Item 10	0.724*
BIS total	0.885*

*p < 0.001.

### 3.6 Subgroup analysis

#### 3.6.1 Gender

Independent *t*-test revealed that there was no difference in the total BIS-M score between male and female cancer participants (mean BIS-M females = 31.71, mean BIS-M males = 30.24, mean difference = −1.472, 95% CI = −3.825 to 0.881, t = −1.233, *p =* 0.219).

#### 3.6.2 Age

One-way ANOVA indicated that there was a significant different in the total BIS-M score between subgroups of age [*F*(3,196) = 2.566, *p =* 0.046]. Post-hoc analysis with Bonferroni correction for multiple testing revealed that those with age of 46 to 65 years (mean difference = −7.941, 95% CI = −15.67 to −0.21, SE = 2.901, *p =* 0.041) and more than 65 years (mean difference = −8.200, 95% CI = −16.41 to −0.01, SE = 3.081, *p =* 0.041) had significantly lower total BIS-M compared with those age 18 to 25 years. However, there was no difference in the score between those age 18 to 25 years and 25 to 45 years.

#### 3.6.3 Education status

One-way ANOVA demonstrated that there was no difference in the total BIS-M score between subgroups of education among the participants [*F*(2,197) = 0.379, *p =* 0.685)].

#### 3.6.4 Religion

One-way ANOVA showed that there was a significant difference in the total BIS-M score between subgroups of age [*F*(3,196) = 2.688, *p =* 0.048]. Post-hoc analysis with Bonferroni correction for multiple testing revealed that those who were Christians had a significantly lower total BIS-M score compared to those who were Buddhists (mean difference = −8.833, 95% CI = −17.14 to −0.53, SE = 3.117, *p =* 0.030). Otherwise, there was no difference in the total BIS-M score between those who were Buddhists and those with other religions.

#### 3.6.5 Time since diagnosis

One-way ANOVA demonstrated that there was no difference in the total BIS-M score between subgroups of time since cancer diagnosis among the participants [*F*(4,195) = 0.045, *p =* 0.996)].

#### 3.6.6 Stage of cancer

One-way ANOVA revealed that there was no difference in the total BIS-M score between subgroups of cancer stage among the participants [F(4,195) = 0.908, *p =* 0.438)].

#### 3.6.7 Types of cancer

One-way ANOVA revealed that there was no difference in the total BIS-M score between subgroups of cancer types among the participants [F(4,195) = 0.453, *p =* 0.770)].

## 4 Discussion

This research aimed to assess the psychometric properties of the BIS-M among cancer patients. The BIS-M demonstrated strong internal consistency (Cronbach’s *α* = 0.895) and good test–retest reliability (ICC = 0.605–0.885). These findings are consistent with those reported in other versions of the Body Image Scale, including the Greek [19], Spanish (20), Turkish (21), Portuguese (23), and Dutch versions (24), which showed similar internal consistency (Cronbach’s α = 0.90–0.96) and test–retest reliability. Assessment of McDonald’s Omega for the BIS-M (ɷ = 0.893) also confirmed good internal consistency (44).

Regarding face and content validity, the study followed standard procedures for translating measurement tools. The original English version of the BIS was translated and back-translated into Malay, reviewed by an expert panel, and subsequently evaluated for content validity. The results showed an S-CVI/Ave of 0.983 and an S-CVI/UA of 0.9, indicating good content validity (45–47). In the pilot study, assessments conducted with native Malay-speaking cancer patients revealed an S-FVI/Ave of 0.983 and an S-FVI/UA of 0.9, confirming good face validity for the BIS-M (32, 47).

CFA confirmed the unidimensionality of the BIS-M items, indicating that a one-factor model provided the best fit, consistent with the original English version of the BIS (14). However, one limitation of the chi-square statistic in CFA is its sensitivity to sample size, as a large sample often results in a statistically significant chi-square (*p* < 0.05). A sample size of 200 or more participants, or a ratio of total subjects to model variables of at least 10:1, is considered adequate for CFA (48). The present study included 200 participants, which may have contributed to the significant chi-square result. Therefore, the chi-square to degrees of freedom ratio is preferred as a more accurate goodness-of-fit indicator than the chi-square statistic alone (48).

The correlations between the BIS-M and the domains and overall score of the BSIQ-SF-M were substantial and favorable, ranging from −0.746 to 0.821 (*p* < 0.001), supporting the convergent validity of the BIS-M (49). These results demonstrate that the BIS-M correlates strongly and consistently with the BSIQ-SF-M, both of which assess perceptions of body image among Malaysian cancer patients. The strong convergent validity supports the coherence and consistency of the Malay version of the BIS (49).

The correlations between the BIS-M items and the overall score of the AAQ-II ranged from 0.001 to 0.151 and were negligibly low, supporting discriminant validity. These findings indicate that body image concerns, as measured by the BIS-M, are distinct from acceptance and psychological flexibility, as measured by the AAQ-II (50). This demonstrates that the Malay version of the BIS effectively differentiates between related but distinct constructs, supporting its discriminant validity (50).

Upon subgroup analysis, the BIS-M was documented to be able to differentiate the degree of body image dissatisfaction between cancer patients with different religions and age, whereby those were Christians had lower body image dissatisfaction compared with Buddhists, while those of increasing age reported lower body image dissatisfaction compared with those of younger age. These findings were in line with existing literature (41, 43). On the contrary, this study did not identify any differences of the subgroups of other demographic and clinical factors suggested to be associated with body image in existing literature, such as gender, education status, stage of cancer, types of cancer and time since diagnosis (41–43). The discrepancies between the study findings and the existing literature could be contributed by methodology and cultural differences.

Although the BIS-M exhibited good psychometric properties, one limitation should be acknowledged. The sample was not representative of the broader Malaysian cancer population, as most participants were female and diagnosed with breast cancer—a group particularly vulnerable to body image concerns due to the visible and identity-related effects of treatment. This demographic concentration may have biased the results and limited the generalizability of the findings to male patients or individuals with other cancer types (e.g., colorectal, prostate, or head and neck cancers). Future research should validate the BIS-M using more diverse and representative samples across genders and cancer diagnoses to enhance its generalizability.

Despite this limitation, the study sample was appropriate for validating the BIS-M. Of the 200 participants, 134 (67%) had undergone surgery, with many being breast cancer patients who may have undergone total mastectomy, while a smaller proportion of head and neck cancer patients may have experienced facial disfigurement following surgery (51). A total of 114 participants (57%) had received radiotherapy, which may negatively affect body image due to adverse effects such as hair loss, skin damage (e.g., scarring and inflammation), and swelling. Additionally, 16 participants (8%) were in stage 4 and had metastases treated with corticosteroids, which can induce weight gain and Cushing’s syndrome features (52, 53). These participants were suitable for evaluating the BIS-M, as they were likely to experience body image concerns.

The BIS-M holds significant practical value in clinical settings. It provides healthcare professionals with a reliable tool to assess body image concerns among Malay-speaking cancer patients, facilitating more individualized psychosocial care. Clinicians can use the scale to identify patients experiencing high levels of body image distress, allowing for timely and targeted therapeutic interventions. Moreover, incorporating the BIS-M into routine assessments enables healthcare providers to monitor changes in body image over time, promoting more responsive care. The quality of this translation and validation study was evaluated using the Consensus-based Standards for the Selection of Health Measurement Instruments Risk of Bias (COSMIN-RoB) checklist, which indicated acceptable quality in assessing internal consistency, test–retest reliability, content and face validity, structural validity, cross-cultural validity, and generalizability.

## 5 Conclusion

This research investigated the psychometric properties of the BIS-M among cancer patients. The results confirmed the BIS-M’s validity, consistency, and reliability. The scale demonstrated good internal consistency, indicating that its items were closely related and accurately reflected concerns about body image. The scale’s construct validity was further supported by factor analysis, which confirmed a unidimensional structure. The BIS-M also showed strong convergent validity, as evidenced by its high correlations with the BSIQ-SF-M. Low correlations between the BIS-M items and the overall AAQ-II score supported its discriminant validity. These findings demonstrate that the Malay version of the BIS is a reliable and practical instrument for assessing body image concerns among cancer patients in Malaysia.

## Data Availability

The raw data supporting the conclusions of this article will be made available by the authors, without undue reservation.
