# Supplementary material for: Translation, adaptation, and validation of the body image scale among cancer patients in Malaysia
Source: Front Public Health. 2026 Mar 24;14:1797807. doi: 10.3389/fpubh.2026.1797807 (PMC13079585; doi:10.3389/fpubh.2026.1797807)
Supplement: Supplementary file 1 [file Supplementary_File_1.docx]

**Supplementary appendix 1. Description of the questions and response options in the socio-demographic and clinical characteristics questionnaire**

Regarding age, the participants were asked the question “What is you age?” and the response options were coded as “18-25 years”, “26-45 years”, “46-65 years”, and “more than 65 years” which follow the increasing trend of age specific incidence of cancer in Malaysia from 2017 to 2021 (Malaysian National Cancer Registry). Regarding gender, the participants were asked the question “What is your gender?” and the response options were coded as “male” and “female.” As for ethnicity, the participants were asked “What is your ethnic background?” and the response options were coded as “Malay,” “Chinese,” “Indian,” and “others.” While for monthly household income, the participants were asked “How much is your monthly household income in Malaysian Ringgit (RM)?” and the response options were coded into “less than RM 6400” (B40), “between RM 6400 to RM 11000” (M40), and “more than RM 11000” (T20) (this coding for monthly household income is according to the Department of Statistics' Malaysia (DOSM) Household Income and Basic Amenities (HIS/BA) survey of 2022 (CompareHero.My Team, 2014). In term of marital status, the participants were asked the question “What is your current marital status?” and the response options were coded as “married” as well as “single/divorced/widow/widower.” For the education status, the participants were asked “What is your highest education attainment?” and the response options were "tertiary education and above," " up to secondary education," and "primary education and below."

Regarding the clinical characteristics, regarding the types of cancer, participants were asked “What types of cancer do you have?” and response options were coded as "breast cancer," "lung cancer," "head and neck cancer," "colorectal cancer," and "others." As for the time since diagnosis, participants were asked “How long have you been diagnosed having cancer?” and the response options were coded as "less than three months," "3-6 months," "6-12 months," "1-2 years," and "more than two years." Finally, as for the stage of cancer, participants were asked “What stage of cancer you are in?” and the response options included "stage 1," "stage 2," "stage 3," and "stage 4". The clinical characteristics were cross-check with the participants case files for confirmation.
